# Analysis of the Price Formation of Agricultural Products and Food in the Agri-Food Chains in Slovenia

**DOI:** 10.3390/foods15101706

**Published:** 2026-05-13

**Authors:** Jernej Prišenk

**Affiliations:** Faculty of Agriculture and Life Sciences, University of Maribor, Pivola 10, 2311 Hoče, Slovenia; jernej.prisenk@um.si; Tel.: +386-2-320-90-00

**Keywords:** agri-food sector, price formation, econometric modelling, Slovenia

## Abstract

The purpose of the article is to present the influences and their weights on the price formation of agricultural and food products in Slovenia. The influences are defined by the ratios of input and output prices and quantities of raw materials, semi-finished products, and products within the food systems of individual stakeholders in the theoretical design of price difference construction, the definition of individual stakeholders’ costs, and the assessment of the dynamics of price and quantity fluctuations from the annual average. The analysis is based on the specified econometric model bases on the Ridge formulation, which represent an analytical model of the price formation in the agri-food chains in Slovenia. The results determine and explain the weight of the impacts based on composite independent variables (based on the calculation of the relationships between individual variables with respect to the mutual responsiveness of changes–elasticity of behaviour) which were defined using available data collected in accordance with the Law on Agriculture in the Republic of Slovenia. Several new independent variables were developed to explain the effects of the independent variable representing the difference in the price of agricultural and food product between the beginning and the end in the analyzed food supply chain. The discussion connects practical actions that address three important future development components of agriculture: strengthening accessibility, competitiveness, and the stability of the position of Slovenian agriculture within the EU.

## 1. Introduction

In the past decade, studies analyzing food systems from a socio-economic perspective have become more frequent [1,2,3]. This type of research is mainly a consequence of rising food prices. An example can also be found in Slovenia, where, according to data from the Statistical Office of the Republic of Slovenia, food prices have risen above the euro area average in the past year. Major changes, often driven by the addition of value by certain actors in agri-food chains, frequently result in higher prices for food products. However, the assessment of whether these price increases are justified is often not within the remit of decision-makers, who must address consumer satisfaction or dissatisfaction. Therefore, analyses that identify and evaluate the underlying reasons for rising food prices are important for all actors directly and indirectly involved in agri-food systems. At the micro level, prices change when the behaviour of consumers and firms change [4]. Analyses of price and market theory are often challenging due to a lack of relevant data, but this has become more feasible following the legal requirement to collect data on the prices and quantities of agricultural products and foodstuffs in Slovenia. Some authors [5] explain that social studies of markets, despite using drastically different terminology, together propose a total of four qualitatively different types of prices: (a) the “quoted price”, which offers some form of rehearsal and reference for a potential market actor regarding what prices they can expect in a market for a given good; (b) the “set price”, which provides information about how much money is offered, or actively demanded, for a certain good; (c) the actual “transaction price”, which is how much a good is actually sold for; and (d) the generalized “compound price”, which offers knowledge about the prices that have been set previously for the same type of good. The research of this study best approaches the third version of the socio-economic market theory that explains price, which we have named “price difference” based on the analyzed data. The collection of data on agricultural or food products in the food supply chain is conducted in accordance with the [6]. The Order specifies all details regarding the collection of data on prices, quantities, and origins of products in the food supply chain, and defines the types of agricultural or food products for which data are collected. As stated in the Instructions for Obliged Parties Submitting Data Based on the Order on Data Collection in the Food Supply Chain, the purpose of the Order [7] is to establish a transparent and clear overview of the situation based on collected aggregate data on purchase and sale weighted prices, quantities, and origins by stakeholder groups in the food supply chain.

Based on the collected data, analyses of the state of the chain are conducted to prepare and implement possible additional timely and targeted measures to support a promising sector or assist it in times of difficulty, particularly to ensure security in the Republic of Slovenia. This also reflects the purpose of this research, which aims to identify the broader impact on the rise in food prices, and present recommendations for achieving the goals of Slovenian agriculture by 2040. The main objective of the research is based on data on prices and quantities in individual sectors and, through the analysis of food price formation, to consequently address the purpose outlined above. For such analyses, researchers often choose between qualitative and quantitative evaluation methods with the econometric approach used in the analysis, as the purpose of the study is to broadly and empirically evaluate agri-food systems to assess the factors that influence the formation of food prices in Slovenia. The purpose of the research is not to determine or evaluate the mark-up shares of individual actors along the food supply chain, but to analyse the areas and identify the causes of the rise in food prices in Slovenia, which can be complex and wide-ranging. An additional challenge, and an originality of the research, lies in the expectation that the results will lead to theoretical and practical conclusions aimed at improving the situation and mitigating the negative causes of rising food prices.

As an added value to the developed theoretical model of food and food product pricing [8], this study analyses the actual price and quantity relationships between producers, processors, intermediaries, and marketers in the Slovenian market that affect the theoretical difference between the price of raw materials and the final (semi-) product on the market. In addition to the costs of production, processing, and preparation for the market, we also considered the broader impacts of exports, imports within the EU, and the market outside the EU area, as well as the dynamics of price and quantity movements during the analyzed period, thus approaching a combination of the economic and social theory of transaction and compound price; this also explain the novelty of the study compared to existing literature. This helps to understand the functioning of individual agri-food chains in the country and to identify weaknesses within the operations of specific actors. Accurate food price prediction can lead to the optimization of resource allocation, increased efficiency, and increased income for the food industry [9,10].

Research on the prices of agricultural and food products is frequent, ongoing, and necessary for each member state of the European Union. Such research is particularly relevant during periods of rising food prices, increasing inflation rates, and the adoption of mitigation measures informed by the results of these analyses. The results are often supported by forecasts and predictions of price movements [11]. Closely linked to this study could be the explanation of the content in study [12] which explains that level of price is determined by the value created by factors of the production process of a commodity and the compensation paid for these factor values. Higher wealth creation typically implies higher value, which may lead to higher costs due to technological innovation, scarcity of resources, highly skilled labor, or a high-quality combination of factors, thereby pushing up the price. If the efficiency of commodity production increases, the same value can be realized at a lower cost, which may reduce the price. The total compensation for the value of factors constitutes the production cost of a commodity. Theoretically, the price is higher than or equal to the production costs of a commodity. The study employed econometric analysis by collecting logarithmic composite variables to examine the forecasted and actual impacts on food product pricing.

Based on the characteristics of the collected data and the additional constraints noted in Section 2.3, we used the Ridge formulation of the econometric model. Ridge regression, also known as Tikhonov regularization, is primarily used to address inverse problems, which arise repeatedly when analyzing situations influenced by factors that are often interdependent and essential for a successful explanation of the problem. This leads to frequent autocorrelation as a systemic issue within certain analyses. For this reason, it was applied in one of the research projects [13,14] which used the Tikhonov fractional regularization method. This method was first proposed in several studies, and the optimality of the order for the partial Tikhonov regularization method was demonstrated in abstract Hilbert spaces [15,16].

Ridge regression is a classic regularization method for linear models that improves stability and predictive performance, particularly in situations with multiple highly correlated explanatory variables and a moderate number of observations. Introducing regularization on the magnitude of the coefficients reduces the variance of the estimates, improves generalization to new data, and allows for more stable estimates even when the X^T^X matrix is nearly singular [17]. In practice, Ridge is often the default choice for the basic stabilization of linear models when the focus is on the prediction and understanding of the direction of effects, rather than the strict selection of a subset of variables.

The paper concludes with a concrete explanation regarding the proposed approaches that should be supported by agricultural policy and can be expressed through the definition of the Vision of Agriculture in Slovenia by 2040 [18].

## 2. Materials and Methods

The chapter on materials and methods is divided into sections on data collection for analysis, background data of the model for analyzing the price formation of food products, the development of a methodological approach for econometric analysis of the price formation model, and the evaluation of the methodological approach.

Before describing the materials and methods used, several hypotheses are presented that guide the entire course of the research and thus shape the methodological approach and methods.

**Hypothesis 1 (H1):** 
*We anticipate that the methodological approach of econometric modelling needs to be adapted to the previous analysis of non-traditional sources of social and economic data [19], collected at the level of data on the prices and quantities of agricultural and food products.*

**Hypothesis 2 (H2):** 
*We anticipate that the results of econometric modelling can be supported and analyzed through expert discussion at the level of general additional data analysis, as well as expert actions at the levels of the production, processing, and marketing of agricultural and food products.*

**Hypothesis 3 (H3):** 
*The research results are expected to enable a broader discussion and the development of recommendations for improving the functioning of agri-food sectors in Slovenia, based on concrete proposals that also address some components of the vision for the development of agriculture in Slovenia by 2040 [18]*.

### 2.1. Data Collection and Selection Procedure

The empirical analysis uses monthly price and quantity data collected from market participants under the national system established by the Law on the Agriculture of the Republic of Slovenia. The dataset includes primary agricultural products and processed food items from the meat, milk, and grain sectors. The types and descriptions of data collected are provided in the source [7], using monthly data for the period July 2024–September 2025. Each month, an average of 6000 data items on purchase quantities and prices, as well as sales quantities and prices of agricultural products and foodstuffs, are reported. This results in a cumulative total of approximately 84,000 data items included in the analysis.

A multi-step data validation procedure was conducted before the econometric analysis. First, weighted average prices were calculated for each product category and reporting period. Individual price observations were then expressed as percentage deviations from the weighted average. Observations exceeding predefined thresholds (+100% or −50%) were flagged as potential reporting errors and subjected to verification procedures. We called this approach a “safeguard moment” which aimed to refine the data received for the first review. Through the “safeguard moment”, approximately 200–300 pieces of data were identified and sent for re-checking on a monthly basis. We had to set suitable limits for detecting the re-evaluation of deviations based on our previous experience with data analysis [8] and with development proceeding in parallel with the cut-off value methodology. A more detailed explanation of the partial contribution of the “safeguard moment” methodology to the definition of the model is provided in Section 2.3. In econometric modelling, a large number of variables can reduce the interpretability of results and diminish model flexibility [20]. Therefore, in the aforementioned study, we included only significant variables: variables y − x8 are important for forming the structure of the theoretical pricing model, as they directly describe the price and quantity relationship between stakeholders; supporting data (x9–x11) determines the costs of individual stakeholders; and the contribution of x12 is described in Section 2.3.

Second, prices and quantities were disaggregated by origin (Slovenia, EU, non-EU). Month-to-month changes were calculated to capture short-term dynamics and to construct relative indicators used in the analytical model. These preparatory steps ensured the internal consistency of the dataset and provided the basis for a higher-level analytical econometric process.

The selected data that were collected for agri-food chain assessment were beef with bone, fresh beef with bone, fresh beef without bone, chicken with bone, fresh chicken with bone, fresh chicken without bone, pork with bone, fresh pork with bone, fresh pork without bone, fresh milk, yogurt, cheese, wheat, flour and bread. The data from Table 1 were defined accordingly. It is worth noting that, due to the impossibility of separating quantities in the dairy sector intended for different types of production, the data were treated uniformly.

### 2.2. Content of Variables of the Developed Model

Table 1 presents the composition of the independent (y) and dependent variables (x1 to x11) that form the model for determining the price of agricultural products and foods.

In-depth analysis of the structure of the model helps us understand transaction cost theory which is defined as the framework that distinguishes between internal and external transaction executions, where internal transactions are coordinated through hierarchy (make-decisions) and external transactions through the market (buy-decisions). Transaction cost theory analyzes the product price in a way which also includes several social activities such as searching for information, and negotiating and enforcing the contracts involved in selling procedures [9]. This could be explained as the “invisible” social relations which are included as influences on y and on dependent variables x1 to x8.

**Table 1:** Description of the content structure of the model.

Variable Label	Variable Description	Role of Variable	Meaning of Variable
Y	Difference (–) between the last selling price in the food chain and the first selling price in the food chain (adjusted, if not the first purchase)	The data shows the ratio between the initial and final prices of an agricultural product or food item in the food supply chain.	The data shows the relationship between input and output prices of agricultural and food products in the food supply chain.
X1	Monthly sales volume of raw material 1 in the food chain (if it is a product, then purchase) divided by the monthly sales of the raw material at the end of the food chain.	The data shows the ratio of input to output quantities in the food supply chain.	Determine the significance of total quantity movements on price formation for agricultural and food products in Slovenia.
X2	Monthly change in selling price 1 in the food chain/monthly change in purchasing price 2 in the food chain	The data illustrates the relationship in price structure between actor 1 and 2 in the food supply chain.	Determine the significance of changes in intersectoral prices in Slovenia on the formation of prices for agricultural and food products in Slovenia.
X3	Monthly change in selling price 2 in the chain/monthly change in purchasing price 3 in the chain	The data illustrates the relationship in price structure between actor 2 and 3 in the food supply chain.	Determine the significance of changes in intersectoral prices in Slovenia on the formation of prices for agricultural and food products in Slovenia.
X4	Monthly change in selling price 3 in the chain divided by the monthly change in purchase price of each subsequent actor in the chain, if it exists	The data illustrates the relationship in price structure between actor 3 and each subsequent item in the food supply chain.	Determine the significance of changes in intersectoral prices in Slovenia on the formation of prices for agricultural and food products in Slovenia.
X5	Repurchase price 1 (and each subsequent) in the SLO chain/Repurchase price 1 in the chain (and each subsequent actor in the chain—maximum 3) EU Depends on the amount of available data Legend: 1-a 2-b 3-c	The data shows the relationship between the prices of agricultural products and foodstuffs in Slovenia and the EU.	Determine the importance of the prices of agricultural and food products in the EU for the formation of the prices of agricultural products and foodstuffs in Slovenia.
X6	Repurchase price 1 (and each subsequent one) in the SLO chain/Repurchase price 1 in the chain (and each subsequent actor in the chain—maximum 3) OUTSIDE EU Depends on the amount of available data Legend: 1-a 2-b 3-c	The data illustrates the relationship between the prices of agricultural products and foodstuffs in Slovenia and outside the EU.	Determine the importance of the price of agricultural and food products outside the EU in the formation of the price of agricultural and food products in Slovenia.
X7	Purchase quantity 1 in the chain (and each subsequent actor in the chain—maximum 3) SLO/Purchase quantity 1 in the chain (and each subsequent actor in the chain—maximum 3) EU Depends on the amount of available data Legend: 1-a 2-b 3-c	The data shows the relationship between the purchase quantities of agricultural products and foodstuffs in Slovenia and the EU.	Determine the importance of imports of agricultural and food products from the EU for the formation of prices for agricultural and food products in Slovenia.
X8	Purchase quantity 1 in the chain (and each subsequent actor in the chain—maximum 3) OUTSIDE EU Depends on available data Legend: 1-a 2-b 3-c	The data illustrates the relationship between the purchase quantities of agricultural products and foodstuffs in Slovenia and outside the EU.	Determine the importance of imports of agricultural and food products from outside the EU for the formation of prices of agricultural and food products in Slovenia.
X9	Data for primary sector [8]	The highest sensitivity to changes in the price of agri-food products occurs when there are changes in cost levels within the primary sector.	Determine the significance of the change in the sensitivity of the primary sector resulting from changes in costs in the formation of agricultural and food products in Slovenia.
X10	Data for secondary sector [8]	The highest sensitivity to changes in the price of agri-food products occurs when there are changes in cost levels within the secondary sector.	Determine the significance of the change in the sensitivity of the secondary sector resulting from changes in costs in the formation of agricultural and food products in Slovenia.
X11	Data for tertiary sector [8]	The highest sensitivity to changes in the price of agri-food products occurs when there are changes in cost levels within the tertiary sector.	Determine the significance of the change in the sensitivity of the tertiary sector resulting from changes in costs in the formation of the prices of agricultural and food products in Slovenia.

### 2.3. “Ridge” Formulation of the Model

The research used the Ridge formulation to analyze the price formation of agricultural and food products with the first difference model. The first difference model was developed based on Durbin–Watson test (DW) calculations for autocorrelation in the model. With this approach, autocorrelation was successfully eliminated, where the DW test values are around two. This approach is justified primarily by the non-stationarity of the dependent variable data, while the independent variables comprise both stationary and non-stationary series. Dividing the model into partially stationary and partially non-stationary levels does not appear reasonable from a purely methodological perspective but rather serves to introduce an appropriate content aspect. In extensive specification models, unified series of either stationary or non-stationary data will never occur. Therefore, we chose to enhance the model by introducing a support variable that assigns a “turbulence” score to each sector, as described in Table 2.

The initial procedure for calculating variable x12 involved determining the ratio of the total periods of mean deviations in the positive and negative directions of individual monthly deviations from the mean or cross-sectional values. This procedure was repeated for both price and quantity calculations. A straightforward calculation, as outlined in Table 2 (column variable description), then followed.

The idea of developing a methodology using the cut-off value stems from the assumption that prices and quantities on the market return to mean or average values in the long term [21] and from upgrading the basic “safeguard moment” approach which is described in Section 2.1 while the maximum value can be understood as the “planted” value and the minimum value as the “harvested” value [22]. The research results are expected to enable a broader discussion and the development of recommendations for improving the functioning of the agri-food sectors in Slovenia based on concrete proposals that also address some components of the vision for the development of agriculture in Slovenia by 2040 [18]. On the one hand, the results indicate that unexplained influences on price fluctuations (deviations from cut-off values) within individual sectors did not significantly affect differences in the prices of agricultural products and foodstuffs in Slovenia; meanwhile, on the other hand the weighting of individual influences on price formation changed with the model upgrade, showing that variable x12 had a significant impact on the overall quality of the improved model results. The formulation of model (1) should therefore be understood as a reciprocal relationship between evaluation processes and additional model formulation processes. Data x5–x8 appeared in versions a, b, and c due to their repetitive structure throughout the agri-food chain. The mathematical formulation of the model structure is as follows:

(1) $$∆y_{t}=\;\beta_{0}+\sum_{i=1}^{k}\beta_{i}∆x_{i,t}+\varepsilon_{t}$$

where

$∆y_{t}$—the first difference in the dependent variable in period t,

$\beta_{0}$—the intercept (constant term) of the model,

$\beta_{i}$—the regression coefficient associated with the i-th explanatory variable,

$∆x_{i,t}$—the first difference in the i-th explanatory variable in period t,

k—the number of explanatory variables included in the model,

$\varepsilon_{t}$—the error term, capturing unobserved influences in period t,

and the extended version of the model:

ΔYt = β0 + β_1_ ΔX_1_t + β_2_ ΔX_2_t + β_3_ ΔX_3_t + β_4_ ΔX_4_t + β_5_ ΔX_5_t + β_6_ ΔX_6_a,t + β_7_ ΔX_6b_,t + β_8_ ΔX_6c_,t + β_9_ ΔX_7a_,t + β_10_ ΔX_7b_,t + β_11_ ΔX_7c_,t + β_12_ ΔX8_a_,t + β_13_ ΔX_8b_,t + β_14_ ΔX_8c_,t + β_15_ ΔX_9a_,t + β_16_ ΔX_9b_,t + β_17_ ΔX_9c_,t + β_18_ ΔX_10_,t + β_19_ ΔX_11_,t + β_20_ ΔX_12_,t + εt; (2)

depends on how many “a-c of x5–x8” exist (see Table 1).

## 3. Results

The results are presented in three subsections: Section 3.1 describes the general characteristics of the developed model; Section 3.2 presents the content part of the model results; and Section 3.3 presents the causal or predictive part of the model results.

### 3.1. Model Adequacy Testing

Choosing the correct value for the regularization parameter (lambda) is crucial in Ridge regression. If lambda is too large, the model becomes overly biased and underfits the data. Conversely, if lambda is too small, the model behaves similarly to a standard linear regression and overfits the data. The model was tested at four different lambda values: 0.01, 0.1, 1, and 10. A lambda value of 10 is considered high, so increasing it further is not justified. Additionally, as the models are estimated using data in first differences, some multicollinearity has already been removed; however, it is not possible to expect that the correlation between variables in the agri-food sector will be completely eliminated, as the prices of agricultural products follow quantities and vice versa (the basic agricultural economic law of supply and demand). This is also reflected in the structure of the variables in the developed model. VIF tests were conducted in all production sectors, highlighting the proportion of coefficients exceeding a value of 10. The highest proportion, 30%, is in the cereal production sector, followed by pork meat at 25% and the dairy sector at 10%. In the other two sectors, no variable showed a high correlation value with the others. In the cereal sector, VIF values are relatively high, which may result from seasonal patterns and common trends among the explanatory variables.

**Figure 1:**
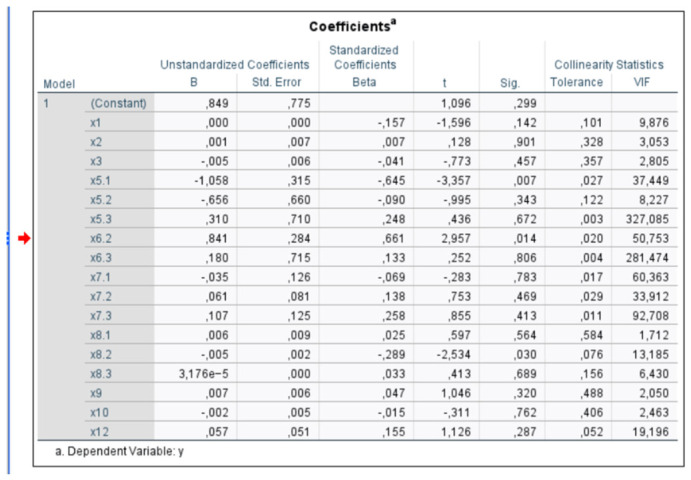
Example of VIF test values for the cereal sector—original printout from the analytical program.

Figure 1 shows the results of the VIF test for the cereal sector, where the VIF values of the variables exceed the threshold value of 10 in 53% of cases and the warning value of 5 in an additional 18%. Although major correlations between variables did not occur in the meat sectors, we chose to use a unified approach to the analysis by applying Ridge regression, following the key takeaway message from both methods:

Ridge regression is particularly useful for addressing multicollinearity by shrinking coefficient estimates and improving model stability. OLS regression can produce unreliable estimates when predictors are correlated, making it less suitable for high-dimensional datasets [23,24].

Therefore, Ridge regression serves primarily as a stabilizing addition rather than the main tool for model correction. The most appropriate model was selected based on a review of the RMSE and MAE tests, which measure forecast errors, and the DW test, which indicates the presence or absence of autocorrelation in the model. It should be noted that higher lambda values result in higher average forecast errors (RMSE—Root Mean Squared Error) and higher average absolute forecast errors (MAE—Mean Absolute Error). The test results for individual lambda values are presented in Table 3, while Table 4 lists, in addition to the test properties of the selected models (tested on lambda 0), the general forms of econometric models for each sector.

An encouraging fact is that, despite the increase in the lambda value, the values of the other criteria and tests do not change significantly, indicating a stable specification of the developed model. If lambda is too high the penalty forces coefficients towards zero too aggressively, leading to severe underfitting (the model becomes too simple). If lambda is small/optimal it slightly shrinks the coefficients—reducing their sensitivity to noise—without losing the underlying trend of the data [24]. Given that greater multicollinearity occurred only in certain sectors, we decided to use the smallest value (0.01).

**Figure 2:**
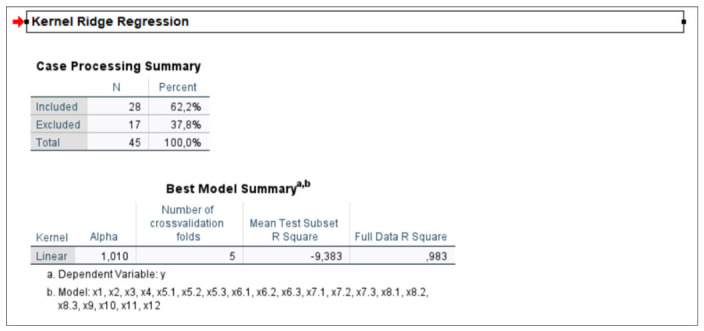
Ridge model properties for the grain sector at a lambda value of 1.010. Original printout from the analytical program.

**Figure 3:**
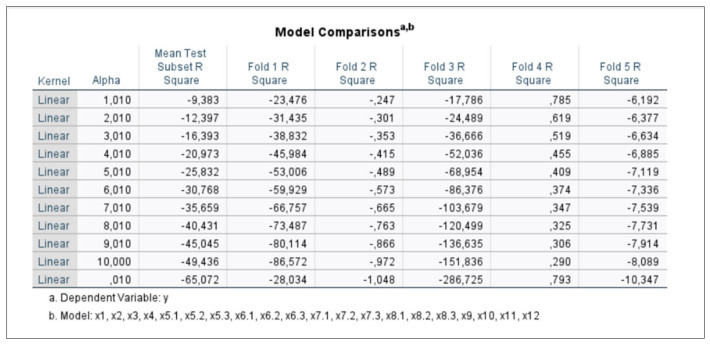
Comparison of the Ridge model for the grain sector in tests ranging from 0.010 to 10. Original printout from the analytical program.

We also examined which lambda values would be most appropriate for the grain sector, where the highest multicollinearity between the data was present. We randomly tested lambda values ranging from 0.01 to 10, with an intermediate test at a value of 1. Figure 2 and Figure 3 show the original results of the grain sector model. Figure 2 explains the properties of the best model, while Figure 3 shows the intermediate tests for different values of lambda (alpha). The best model was selected based on a lambda value of 1.01.

### 3.2. Description of Content Part of the Model Results

For the developed model, the data were used in standardized form, so it was necessary to convert the standardized Ridge estimated coefficient back into a form suitable for calculating elasticity according to a linear functional form. Positive elasticity values were converted into a percentage of the weight of each individual data item. For data groups x5–x8, where the number of sub-data items (a–c) differed, the elasticity values were previously combined by summing them. The calculated weights indicate the importance of individual data, reflecting their impact on the formation of prices for agricultural and food products (Table 5).

Table 6 provides a more detailed overview of the first three impacts on individual production sectors that most significantly influence the formation of prices for agricultural and food products. All other data had a minor impact on the price formation of agricultural and food products by sector (the sum of all others accounts for the difference to the total weight value of 100%).

### 3.3. Prediction Values of the Model

When presenting forecasts based on the results of the “Ridge” model, both the accuracy of the forecast values and the direction indicated by the sign of the forecast, which determines future movement, are particularly important. The results show the predicted price movements of agricultural products and foodstuffs across individual chains. It should be noted that the so-called “tails” of the curves, representing data from the last month or two, may be unreliable, as forecast accuracy decreases over time. Therefore, it is important to have a longer future time period available (and thus a larger database for training the model). Additionally, the safety margin within the last two months is not included. Consequently, when calculating the model forecast, we excluded the last two months from the period considered, and the forecast data are presented in Table 7. Mean absolute percentage error measures the average magnitude of error produced by a model.

The forecasts for the dairy sector are therefore the least accurate, with an estimated difference of approximately 11.5%. Accuracy estimates for all other sectors are closer. A slowdown is expected across all sectors in the future based on price differences across chains or smaller price increases.

## 4. Discussion

The three main factors influencing the price of agricultural and food products in Slovenia, in order of significance, are:

The importance of the price of agricultural and food products in the EU > the impact of changes in the sensitivity of the primary sector, resulting from cost changes > and the importance of imports of agricultural and food products from the EU.

All other factors were given a lower weighting. However, this weighting varied slightly between individual sub-sectors, with the results shown in Table 6.

If we compare only the three most influential factors with the highest weighting, the differences are evident in that in the chicken meat production sector input costs at the primary and tertiary levels play a significant role in price formation, which is consequently reflected in fluctuations in imports of agricultural and food products. In the case of beef and pork production, it is similarly notable that the price of agricultural products and foodstuffs from the EU have a significant impact on price formation in both sectors. It is also worth noting that for pork, the significance of price changes at the cross-sectoral level in Slovenia appears as an individual factor only in the pork production sector. This is understandable, as the purchase prices of live animals change more rapidly and intensively than in the other two sub-sectors.

In the analysis of pork meat, the econometric model accurately identified the external impact of food prices at the EU level, as the purchase prices of live animals in Slovenia are often based on the stock exchange price from Austria for 58% slaughter without mark-ups.

In the dairy sector, the prices of raw materials and products responded most significantly to changes in milk imports from outside EU member states and to changes in costs at the milk production stage for producers. Together, these two factors account for as much as 75% of all reasons for price formation in this sector (Table 6). This claim was further verified through a descriptive analysis of the quantities of processed dairy products for which data are collected, as presented in Table 8 and Table 9. The data confirm the analytical results of the model in both cases, except for cheese purchases from retailers, where no significant deviation from the quantities in 2024–2025 is observed.

**Figure 4:**
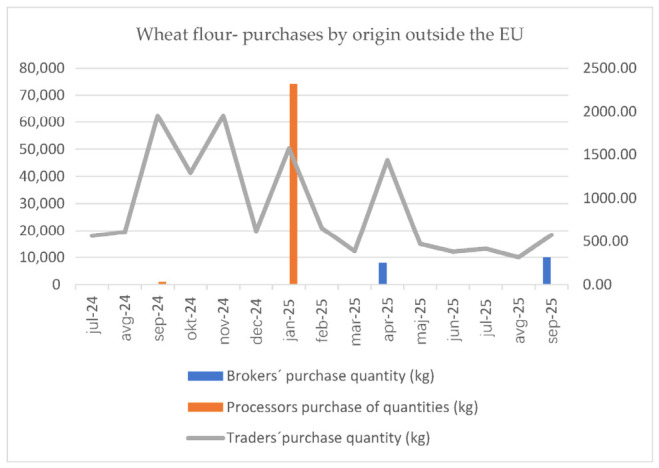
Graphical illustration of wheat flour purchases by origin outside the EU (July 2024–September 2025).

**Figure 5:**
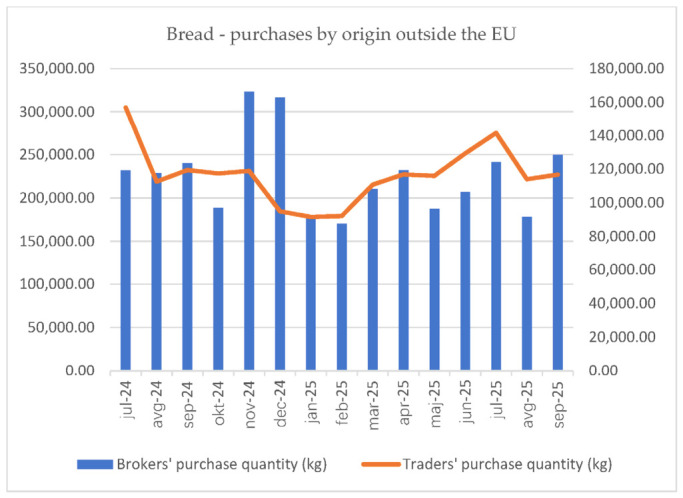
Graphical illustration of bread purchases by origin outside the EU (July 2024–September 2025).

In the grain sector, price formation depends primarily on the supply of raw materials of Slovenian origin. If this supply is insufficient, imports of finished products are triggered at the trade stage. The impact of imports is most significant at the level of flour and bread, with a significance ratio of 1:2.75. The descriptive analysis illustrates the above with Figure 4 and Figure 5.

Generally, the factors influencing the price formation of agricultural and food products in Slovenia are distributed along agri-food chains, including related EU and non-EU market factors. It is important to emphasize that the two main factors influencing the price difference are the rapid response of actors to increased domestic production costs and the efforts of end providers to balance sufficient food quantities on the market by replacing imports, primarily from EU member states.

As part of the research, additional descriptive analyses of the price list for agricultural and food products were conducted across three periods. From July 2024 to December 2025, price increases were observed in almost all segments of the agri-food chains. In all sectors, prices for agricultural products and semi-finished products rose during the production and processing phases, while they decreased at the retail level. The price of final products such as bread increased during the trade phase. In both cases, these fluctuations in the grain sector were 3–5%. Larger price fluctuations were observed in the beef sector, where the purchase price at producers increased by 34%, while the price of fresh packaged meat at retailers rose by approximately 10%. In the meat processing sector, the purchase price of raw milk did not follow the prices of milk, yoghurt, and cheese on store shelves. In some segments, this difference was as much as 15%. Such descriptive analyses are useful, but they do not help in understanding the factors influencing price formation; rather, they provide a basis for further research, as described in the conclusion of the paper, with the possibility for further development in the area of price transmission along agri-food chains.

The results align with the basic agrarian economic theory of market demand and supply [25], as indicated by x5, x6, and x7 (across all sectors), and in certain segments, they reflect the impact of costs on price formation, as shown by x9 in the meat sector. However, the research findings can also be partly associated with modern economic theory (which states that the modern economy is based on innovation, knowledge, and technology [26]), which is fully integrated into the described relationships between stakeholders that influence the price formation of agricultural and food products, as defined by variables x1–x4 (Table 10).

Based on the results of the study, existing knowledge in the field can be confirmed and summarized as follows:

- The Slovenian market for agricultural products and foodstuffs is highly responsive and, due to its small size and sensitivity to the cost dynamics of production, processing, and marketing, is susceptible to intended interventions.
- In addition to cost sensitivity, the economic and social characteristics of the primary agricultural sector are also expected to influence the dynamics of price and quantity fluctuations in agri-food chains.
- A normal response has occurred in ensuring supply and forming the price of agricultural products and foodstuffs on the Slovenian market through imports from the EU when production, processing, and marketing respond negatively to increased costs.
- It cannot be claimed that any actor in the agri-food chains intervened in price formation for agricultural products and foodstuffs during the analyzed period to such an extent that it visibly affected the analysis results. Nevertheless, it is important to highlight the rapid response of all participants to changes in costs.
- The EU market determines the price of agricultural products and foodstuffs and partly influences quantities, while origins outside the EU do not have a significant impact on the Slovenian market.

## 5. Conclusions

In the final chapter, we present a structured response to the hypotheses raised. At the same time, all existing recommendations present challenges for future research.

We assume that introducing a support variable to describe the “turbulence” of individual sectors did not significantly contribute to price formation, partly due to the successful implementation of a “safeguard moment” during the refinement of basic data. Based on the developed approach, the theoretical design of the “safeguard moment” can be defined as follows:

“The ‘safeguard moment’ approach in data analysis in agri-food chains is decisive and robust for data collection at the macro level, and restrained and selective at the micro level of analysis in agri-food systems.”

The inclusion of x12 in the estimation of price and quantity fluctuations affected the model as a whole, although x12 did not contribute significantly to the pricing of agricultural and food products on its own. The vast majority of other data representing the effects on pricing have changed. The truncated Table 10 presents the changes in the effects of individual variables on pricing, while the previous data without the inclusion of x12 in the model are shown in brackets.

By pre-processing the data and including specific variables, we ensured that the theoretical basis for different market price terminologies was followed while capturing the specifics of agri-food systems, as reflected in the final results of the model. We therefore anticipate that we can confirm Hypothesis 1.

The formation of the purchase price of milk for producers is driven primarily by milk production costs (thus, we cannot confirm that the purchase prices of milk are tracking production costs). Therefore, it is necessary to address this issue with additional analysis of the competitiveness of individual agricultural holdings at the level of production volume in Slovenia. The descriptive analysis presented in the discussion section supports Hypothesis 2 regarding the possibility of verifying the modelling results through the expert discussion of the descriptive analyses of data collected at the level of agri-food systems in Slovenia.

The research results can support the conclusions and address the measures aimed at the vision for Slovenian agriculture until 2040, as stated below:

(1) In the beef sector, data on the importance of the price of agricultural and food products from the EU for price formation in Slovenia showed a significant weighting in the final results of the model. From this, we can conclude that quantities have an indirect impact on food price formation in Slovenia. The origin of agricultural and food products in the food supply chain data collection order defines the origin at the processing and preparation stage, while the preparation stage for the market is not considered for the purpose of demonstrating origin. However, quality labels also cover this latter stage through traceability [7].

PROPOSAL FOR PRACTICAL ACTION: “Obtain information based on produced and processed quantities of raw materials that demonstrate Slovenian origin through quality labels.” The measure addresses the accessibility, competitiveness, and stability of agriculture in Slovenia.

(2) In the dairy production sector, it is necessary to understand the rapid adjustment of production costs to changes in the purchase price of milk, which primarily addresses the components of competitiveness and stability.

PROPOSAL FOR PRACTICAL ACTION: “Conduct additional analysis or research on the impact of costs on the assessment of production competitiveness in the dairy production sector in relation to the quantity production of farms in Slovenia.” This measure primarily addresses the competitiveness and stability components of the vision for agriculture in Slovenia.

(3) In the cereal sector, the price formation of final products depends on the movement of the quantities of (bread) grains produced in Slovenia.

PROPOSAL FOR PRACTICAL MEASUREMENT: “Address the declining interest in sowing bread grains through agricultural policy measures.” This measure primarily addresses the stability component of the vision for agriculture in Slovenia. Hypothesis 3 can be confirmed.

This study offers a new perspective on understanding the pricing of agricultural products and food in Slovenia, a small market with relatively diverse farming opportunities. It adds value by explaining the demonstrable impact of developments within the EU and outside the EU area. The study has the potential for further development by analyzing the calculated sensitivity of price changes to various increases in agricultural input costs. This would enable the identification of practical and precautionary measures to protect the most vulnerable consumer groups, while ensuring they have access to quality food produced in Slovenia. A limitation of this study lies in the treatment of data for analysis, as the data were handled as strictly separate sets obtained at the level of Slovenia, the EU market, and the non-EU market, as already explained under point 1 of the analysis of Hypothesis 3. Special possibilities for developing the study in future include analyzing price transmission by actors along agri-food chains.

## Figures and Tables

**Table 2:** The description and results of the impact of variable x12 on the model outcomes.

Variable Label	Variable Description	Role of Variable	Meaning of Variable
X12	The deviation of price from the observed cross-sectional value of zero towards the minimum or maximum. The deviation of quantity from the observed cross-sectional value of zero towards the minimum or maximum.	The data shows the relationship between fluctuations in food prices and fluctuations in quantities within the chain.	Evaluate the impact of the so-called “turbulence” in an individual sector during the observed period on the formation of food prices.

**Table 3:** Results of the econometric model based on different values of lambda and various production sectors.

Sector	Lambda (λ)	RMSE	MAE	R2	Adj_R2	DW
Beef sector	0.01	0.60	0.46	0.63	0.29	2.0
Beef sector	0.10	0.60	0.46	0.63	0.29	2.0
Beef sector	1.00	0.60	0.46	0.63	0.29	2.0
Beef sector	10.00	0.62	0.47	0.61	0.26	2.0
Pork sector	0.01	0.60	0.46	0.63	0.29	2.1
Pork sector	0.10	0.60	0.46	0.63	0.29	2.1
Pork sector	1.00	0.61	0.47	0.62	0.28	2.1
Pork sector	10.00	0.67	0.50	0.54	0.12	2.1
Chicken sector	0.01	0.61	0.42	0.62	0.27	2.2
Chicken sector	0.10	0.61	0.42	0.62	0.27	2.2
Chicken sector	1.00	0.62	0.42	0.61	0.26	2.2
Chicken sector	10.00	0.67	0.46	0.54	0.13	2.2
Milk sector	0.01	0.21	0.16	0.95	0.91	2.0
Milk sector	0.10	0.21	0.16	0.95	0.91	2.0
Milk sector	1.00	0.23	0.17	0.94	0.89	2.2
Milk sector	10.00	0.32	0.23	0.90	0.80	2.5
Cereal sector	0.01	0.25	0.17	0.94	0.88	2.6
Cereal sector	0.10	0.25	0.17	0.94	0.88	2.7
Cereal sector	1.00	0.26	0.18	0.93	0.87	2.6
Cereal sector	10.00	0.34	0.24	0.88	0.77	2.5

**Table 4:** Estimation results and model formulation for several production sectors.

Sector	Lambda (λ)	RMSE	MAE	R2	Adj_R2	DW	Model Formulation
Beef sector	0.01	0.60	0.46	0.63	0.29	2.0	$∆y_{t}^{BS}=\beta_{0}^{BS}+\sum_{i=1}^{k_{BS}}\beta_{i}^{BS}∆x_{i,t}+\varepsilon_{t}^{BS}$
Pork sector	0.01	0.60	0.46	0.63	0.29	2.1	$∆y_{t}^{PS}=\beta_{0}^{PS}+\sum_{i=1}^{k_{PS}}\beta_{i}^{PS}∆x_{i,t}+\varepsilon_{t}^{PS}$
Chicken sector	0.01	0.61	0.42	0.62	0.27	2.2	$∆y_{t}^{CS}=\beta_{0}^{CS}+\sum_{i=1}^{k_{CS}}\beta_{i}^{CS}∆x_{i,t}+\varepsilon_{t}^{CS}$
Dairy sector	0.01	0.21	0.16	0.95	0.91	2.0	$∆y_{t}^{ML}=\beta_{0}^{ML}+\sum_{i=1}^{k_{ML}}\beta_{i}^{ML}∆x_{i,t}+\varepsilon_{t}^{ML}$
Cereal sector	0.01	0.25	0.17	0.94	0.88	2.6	$∆y_{t}^{CR}=\beta_{0}^{CR}+\sum_{i=1}^{k_{CR}}\beta_{i}^{CR}∆x_{i,t}+\varepsilon_{t}^{CR}$

**Table 5:** Results of econometric model for assessing impact on price formation of agricultural and food products in Slovenia.

Variable	Meat Sector	Cereal Sector	Dairy Sector
y	/	/	/
x1	3	53	0
x2	5	0	0
x3	2	0	0
x4	0	0	0
x5	35	1	4
x6	5	2	1
x7	15	3	4
x8	3	32	44
x9	20	0	32
x10	5	4	6
x11	5	4	9
x12	1	0	0

**Table 6:** Description of the weighting values of the three main influences on the price formation of agricultural and food products by sector.

Sector	Type of Data and Weight Values	Type of Data and Weight Values	Type of Data and Weight Values
Chicken meat	x9	x7a	x11
Weight value	44%	13%	10%
Beef meat	x5a	x6b	x7c
Weight value	54%	9%	7%
Pork meat	x5c	x7b	x2
Weight value	28%	23%	14%
Cereal sector	x1	x8c	x8b
Weight value	53%	22%	8%
Dairy sector	x8c	x9	x11
Weight value	43%	32%	9%

**Table 7:** Forecast data for individual production sectors for the period July 2024 to July 2025.

Type of Observation	Beef Sector	Pork Sector	Chicken Sector	Dairy Sector	Cereal Sector
Average error deviation between actual and forecast values	−0.033	−0.006	−0.008	0.003	−0.004
MAPE value	5.71	4.27	0.4	11.53	4.85
Average forecast value in observed period	0.06	0.04	0.004	0.11	0.05

**Table 8:** Purchase of yogurt by origin outside the EU for the period July 2024 to September 2025.

Yoghurt Purchase (Origin OUTSIDE EU)		
Period	Brokers’ Purchase Quantity (l)	Traders’ Purchase Quantity (l)
jul.24	59,839.95	158.69
avg.24	44,026.04	241.89
sep.24	73,071.63	136.50
okt.24	96,630.45	284.70
nov.24	75,984.30	214.80
dec.24	82,752.46	157.47
jan.25	60,781.69	130.05
feb.25	95,775.88	121.47
mar.25	66,846.82	130.12
apr.25	101,653.35	126.44
maj.25	79,943.65	228.90
jun.25	132,778.80	239.40
jul.25	79,751.69	325.80
avg.25	73,374.60	288.60
sep.25	97,063.18	312.15

**Table 9:** Purchase of cheese by origin outside the EU for the period July 2024 to September 2025.

Cheese Purchase (Origin OUTSIDE EU)		
PERIOD	Brokers’ Purchase Quantity (kg)	Traders’ Purchase Quantity (kg)
jul.24	281.00	5111.49
avg.24	264.50	10,866.36
sep.24	182.00	11,708.31
okt.24	270.00	6592.41
nov.24	204.00	6830.70
dec.24	405.00	9441.47
jan.25	667.00	5256.03
feb.25	476.00	5822.92
mar.25	239.50	7546.60
apr.25	718.00	3805.12
maj.25	689.00	722.04
jun.25	812.00	1364.68
jul.25	644.00	2043.27
avg.25	955.00	1320.48
sep.25	687.40	904.00

**Table 10:** Estimated impacts of individual variables on the pricing of agricultural products and food before (data are presented in brackets) and after including an x12 in the model.

Variable	Meat Sector	Cereal Sector	Dairy Sector
y	/	/	/
x1	3 (8)	53 (0)	0 (0)
x2	5 (0)	0 (3)	0 (0)
x3	2 (0)	0 (0)	0 (0)
x4	0 (0)	0 (0)	0 (0)
x5	35 (52)	1 (50)	4 (52)
x6	5 (7)	2 (17)	1 (17)
x7	15 (11)	3 (18)	4 (6)
x8	3 (1)	32 (4)	44 (4)
x9	20 (11)	0 (5)	32 (3)
x10	5 (0)	4 (4)	6 (2)
x11	5 (10)	4 (2)	9 (4)
x12	1	0	0

## Data Availability

The original contributions presented in the study are included in the article, further inquiries can be directed to the corresponding author.
